# Strømme syndrome: the clinical and molecular spectrum associated with variants in *CENPF*

**DOI:** 10.1038/s41431-026-02187-7

**Published:** 2026-07-18

**Authors:** Meghan R. Mulligan, Heather Mulhall, Samuel R. A. Hawarden, David Davies-Payne, Raoul Heller, Louise S. Bicknell

**Affiliations:** 1 Department of Biochemistry, University of Otago, Dunedin, New Zealand; 2 William Harvey Research Institute, Queen Mary University of London, London, UK; 3 Department of Radiology, Starship Children’s Hospital, Auckland, New Zealand; 4 Genetic Health Service NZ - Northern Hub, Te Toka Tumai Auckland, Auckland, New Zealand

**Keywords:** Disease genetics, Medical genetics

## Abstract

Strømme syndrome (MIM 243605) or a form of syndromic microcephaly have both been reported to be caused by biallelic variants in *CENPF*. The common features associated with *CENPF* variants are microcephaly, jejunal atresia and eye abnormalities, but additional case reports have indicated more variable presentations. Here, we describe the diagnosis of an adult with Strømme syndrome and present a review of all 32 cases reported with genetic variants in *CENPF*, to highlight the common and diverse features. We find that neurological abnormalities, particularly microcephaly, are the most common feature (91% of individuals), with gastrointestinal (69%) and ocular features (44%) less common. While facial dysmorphism is widely reported, there are a broad variety of features noted, with no clear commonalities. The vast majority of genetic variants identified alter the reading frame through either nonsense, frameshift, or more rarely, essential splice site variants. Missense variants are seldomly reported; all are homozygous in affected individuals, but functional evidence has been presented for only one missense variant. Our summary illustrates the breadth of clinical features associated with Strømme syndrome caused by *CENPF* variants and the molecular genetics of *CENPF* reported thus far.

## Introduction

Correct chromosomal segregation is vital for cell division in both development and health. This segregation is driven by the formation of the mitotic spindle, a protein complex derived from the centrosome that anchors microtubules for chromosome spreading, and the kinetochore, a chromosome-attached complex that forms at the centromeres to tether chromosomes to microtubules. Reduced functionality of these processes is a common driver of both developmental and somatic-based disease (primarily cancer), with microcephaly (reduced brain size) being a common developmental consequence of inaccurate positioning and efficiency of these mitotic processes [1].

*Centromere Protein F (CENPF)* is a 20-exon gene on chromosome 1 that encodes a large (3114 amino acid) coiled-coil protein which functions as a homodimer and is required for kinetochore function and cell-cycle progression. Multiple functions have been ascribed to CENPF, likely because of the many protein-protein interactions identified thus far, including many that occur in a cell-cycle-specific manner [2, 3]. CENPF is involved in chromosome segregation, kinetochore assembly and activity during mitosis [4–6]. Within the kinetochore, through interacting with Bub1, it functions in the fibrous corona section of the outer kinetochore complex, where the protein serves as a platform for regulated protein binding between kinetochore and microtubules [5–7]. CENPF moves from kinetochores to the spindle midzone in early anaphase and then during cytokinesis, helps traffic mitochondria to daughter cells [8]. CENPF also has apparent nuclear roles including association with the nuclear envelope (interacting with Nup133) and changes in gene expression [2, 4].

Strømme syndrome (MIM 243605), a form of syndromic microcephaly, displays autosomal recessive inheritance and has been reported to be caused by biallelic variants in *CENPF* [9, 10]. With subsequent case reports, the common features associated with *CENPF* variants are microcephaly, jejunal atresia and eye abnormalities, but different case reports have indicated more variable presentations [9–22]. CENPF-related disorders are generally considered to be ciliopathies: the protein localises to the basal bodies of primary cilia, interacts with IFT88, and reduced expression in a zebrafish model produces canonical ciliary phenotypes, including laterality defects, hydrocephalus, and kidney abnormalities [10]. Separating out whether developmental consequences are the result of abnormal mitosis or ciliogenesis defects requires further investigation.

Here we describe an adult with the diagnosis of Strømme syndrome and present a review of all 32 cases reported with genetic variants in *CENPF*, to highlight the common and diverse features (Supplementary Table 1) [9–22]. We find that neurological abnormalities, particularly microcephaly, are the most common feature (91% of individuals), with gastrointestinal and ocular features less common. While facial dysmorphism is widely reported, there are a broad variety of features noted, with no clear commonalities. Our summary illustrates the breadth of clinical features associated with Strømme syndrome caused by *CENPF* variants and the molecular genetics of *CENPF* reported thus far.

## Methods

Identification of *CENPF* variants in New Zealand patient: Ethics approval was provided by the New Zealand Health and Disability Ethics Committee (16/STH/3). DNA from the New Zealand proband and both parents were subjected to whole exome sequencing using the Agilent V7 capture kit, followed by sequencing on an Illumina HiSeq 4000. Sequencing data were processed by a standard GATK-based pipeline as previously described [23]. Given the consanguinity present in the family, and the common biallelic inheritance of variants associated with microcephaly, recessive inheritance or compound heterozygous inheritance models were prioritised. Sanger sequencing was undertaken to confirm segregation of *CENPF* variants in the three family members. Amplification of DNA and sequencing was undertaken using oligonucleotides CENPF_variants_F: GCTCAGCAGGACCTCAATCT

CENPF_variants_R: GCAGGCTTTCAGATTCCTTGAC, using methodology previously described [23]. Both variants were sequenced using the same PCR oligonucleotides.

Sequence alignment: Clustal Omega was used to align CENPF amino acid sequences from different organisms using the following uniprot identifiers: *Homo sapiens* P49454, *Pan troglodytes* H2Q149, *Mus musculus* E9Q3P4, *Gallus gallus* A0A8V0Z9Z7, *Danio rerio* F1Q7X5, *Xenopus laevis* A0A974CTG9.

## Results

### Proband clinical presentation and genetic analysis

A family with an affected male proband with microcephaly and intestinal atresia was recruited for exome sequencing analysis to identify the causative variants. The healthy parents of Persian speaking Afghani ancestry were 5th degree relatives to each other (second degree cousins). Their family history was otherwise unremarkable.

The patient was naturally conceived and born prematurely at 35 weeks with a birth weight of 1985g (11th centile). He had a congenital omphalocele and duodenal atresia. He underwent surgical repair and subsequent revision surgery (side-to-side duodenojejunostomy) for recurrent complications and temporarily required total parenteral nutrition with pancreatic enzyme replacement therapy for exocrine pancreatic insufficiency. A faecal elastase level recorded at 3 weeks of age corrected for prematurity was 21 µg/g wet faeces (reference range >200 µg/g). His stool chymotrypsin normalised around the age of 15 months when he was gradually weaned of pancreatic enzyme replacement. His gastrostomy was finally closed at the age of 3 years and 10 months.

Primary microcephaly was noted in infancy. A brain MRI at the age of almost 2 years revealed dilatation of the occipital horns and partial agenesis of the corpus callosum involving the rostrum and splenium. White matter volume was reduced, but signal characteristics were unremarkable. Auditory brain stem evoked potentials and full ophthalmic examination were normal.

Early paediatric assessment documented subtle dysmorphic features including a prominent nose, slightly down-sloping palpebral fissures and large low set ears. His measurements at 24 weeks (19 weeks corrected for prematurity) were: occipitofrontal head circumference = 32.0 cm (7.5 cm below P0.4), height = 55.8 cm (3 cm below P0.4), weight = 4.1 kg (1.2 kg below P0.4).

Neurodevelopmental progress was mixed. Social smiling was observed from 6 weeks of age. At 25 weeks of age corrected the proband had adequate fine motor skills, reaching out and grabbing hold of a spatula. He was able to transfer objects from one to the other hand. He was weight-bearing and able to hold his head without obvious head lag when pulled to sit. At 13 months of age, he was able to pull to stand and cruised around furniture, and at 21 months he was running and climbing. There was significant speech delay and learning difficulties. Raised in a bilingual environment he did not have single words in English until after 2.5 years of age. At the age of 5 years he succeeded to put together 2–3 word sentences in English. At the age of 5 years and 6 months his language and problems solving skills were at the level of a 2–3-year-old per Griffiths Mental Developmental Scales. A formal diagnosis of intellectual disability was confirmed at the age of 20 years (FSIQ 45–53, 95% CI WAIS-IV A&NZ).

**Fig. 1:**
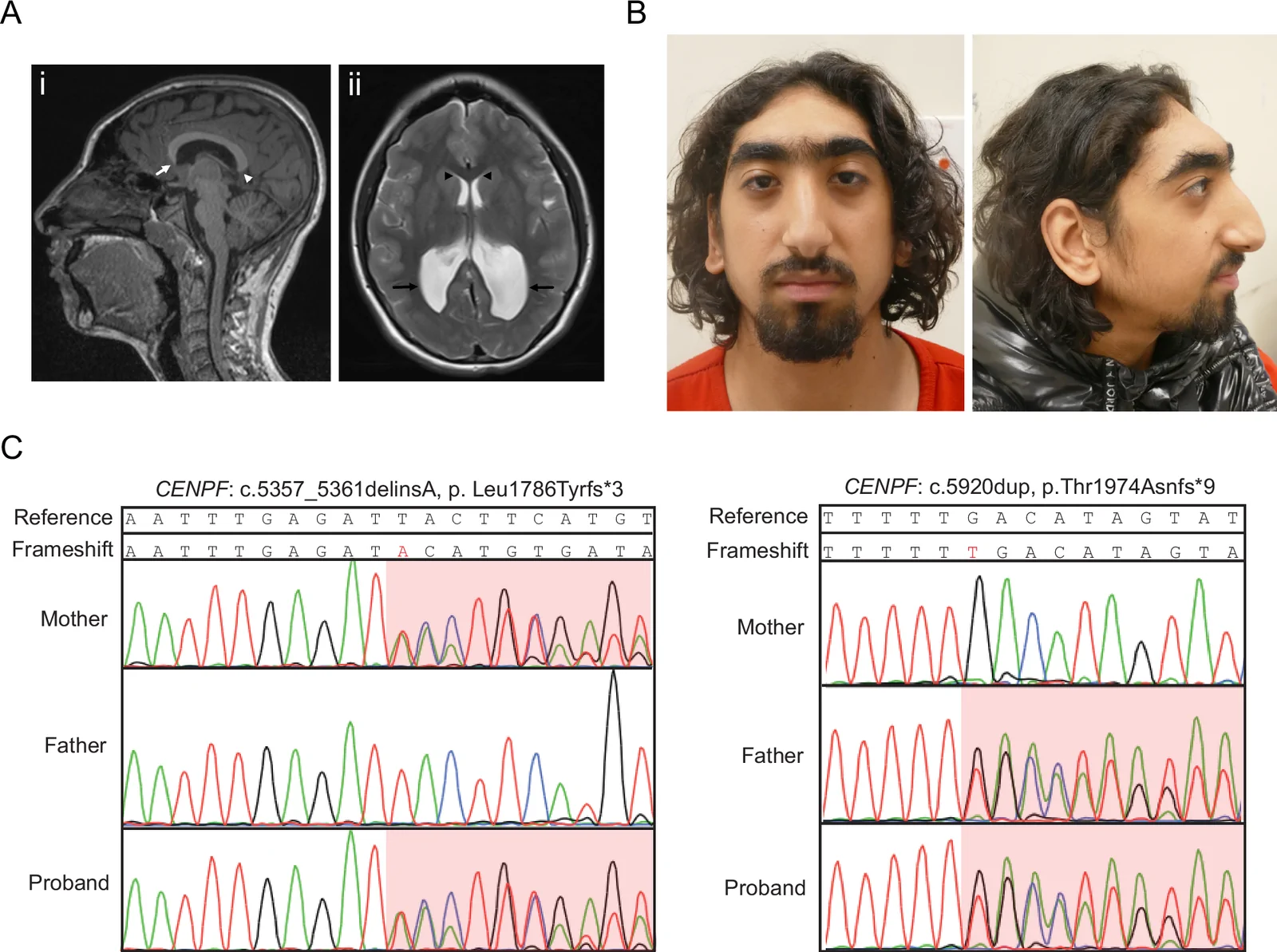
New Zealand patient with variants in *CENPF.* **A** MRI at 11 years of age. Ai: The sagittal T1 image shows microcephaly, with reduced frontal lobe size. The corpus callosum shows hypoplasia of the rostrum (arrow) and splenium (arrowhead). Aii: The axial T2 image demonstrates enlarged atria and occipital horns (arrows) with reduction of the adjacent white matter. The ventricular frontal horns (arrowheads) are not dilated, with normal white matter volume adjacent. The frontal lobes are small, with gyral simplification. **B** Facial photos of the proband, showing - in addition to obvious microcephaly -subtle dysmorphisms with bushy eyebrows and synophrys, a prominent nose, large and low set ears and an elongated shape of the face with pointed chin. **C** Sanger sequencing confirming the segregation of two variants in *CENPF* (NM_016343.4): c.5357_5361delinsA, p. Leu1786Tyrfs*3 and c.5920dup, p.Thr1974Asnfs*9.

Neurobehavioural issues emerged in late childhood/teenage years. The patient received a formal diagnosis of Attention Deficit Hyperactivity Disorder at the age of 10 years. Impatient and oppositional behaviour became increasingly challenging from school age on, and an anxiety disorder was aggravated by a history of traumatic experiences at school throughout teenage years that eventually required a change of environment. He was treated with risperidone. An MRI at age 11 showed microcephaly, with reduced frontal lobe size. The corpus callosum shows hypoplasia of the rostrum and splenium (Fig. 1Ai). The axial T2 image showed enlarged atria and occipital horns with reduction of the adjacent white matter. Ventricular frontal horns were not dilated, with normal white matter volume adjacent. The frontal lobes are small, with gyral simplification, and a smaller pons is observed (Fig. 1Aii).

In terms of physical health and wellbeing the patient developed thoracic kyphosis. Significant chronic musculoskeletal pain (knees, shoulders, spine) prompted spinal MRI at the age of 19 years with no cause identified. A diagnosis of central pain sensitisation was made [24]. At his most recent physical exam at the age of 21 years (Fig. 1B), the patient presented with microcephaly (46.5 cm, 7 cm <P3; parental head circumferences of 56.5 cm (father) and 56.0 cm (mother), respectively), short stature (157 cm 5.5 cm < P3; parental heights 156 cm for mother and 179 cm for father; estimated midparental target height = 174 cm) and BMI of 18.66 kg/m^2^ corresponding to P3-P10. (KiGGS 2011 centile charts were used for approximation in the absence of widely accepted WHO centile charts for head circumference beyond 5 years of age [25]). He has a long face with pointed chin, bushy eyebrows with synophrys, a prominent nasal root and slender nasal dorsum with prominent columella. He has large low set ears and was diagnosed with mild conductive unilateral hearing loss. He complained about tinnitus on his right ear. His renal tract appeared normal on ultrasound. In the absence of any clinical findings suggesting a heart defect he has never had a cardiac ultrasound. Baseline laboratory investigations including liver, renal, and thyroid function tests returned normal results. A molecular karyotype (Illumina Infinium Global Screening Array – 24 v3.0 with a resolution of 0.2 Mb) showed a normal male karyotype without any clinically significant copy number change, but with several long continuous stretches of homozygosity >5 Mb on chromosomes 4, 5 and 12, representing about 1.8% of the genome. This was compatible with the known 5th degree of parental consanguinity [26].

Following sequencing and variant prioritisation, genetic analysis identified that the patient was compound heterozygous for variants in *CENPF* (NM_016343.4): c.5357_5361delinsA, p.(Leu1786Tyrfs*3) (GRCh37 chr1:g.214818270_214818274delinsA) (CADD: not computed, gnomAD v4.1: not present) and c.5920dup, p.(Thr1974Asnfs*9), (GRCh37 chr1:g.214818833dup) (CADD: 25.3, gnomAD v4.1: groupmax allele frequency 0.00006787). Sanger sequencing confirmed the presence of both variants in the patient, and that each parent is a carrier for one variant (Fig. 1C).

### Molecular summary

**Fig. 2:**
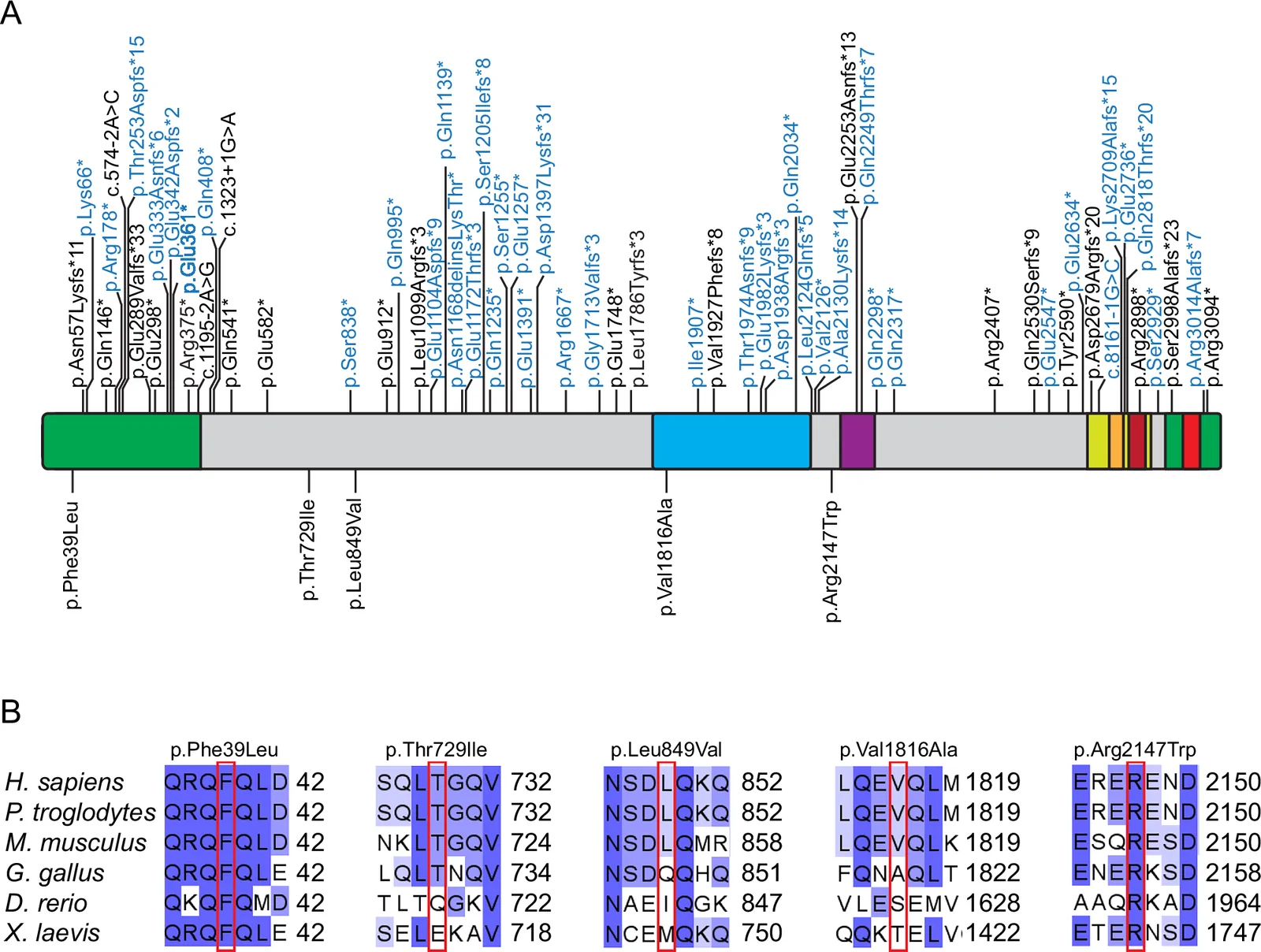
Genetic variants identified in CENPF. **A** Protein or transcript level variants identified in either the literature (referenced within the text) or ClinVar (blue, accessed November 2025). Truncating variants are above the protein schematic, missense variants below. Notable protein regions coloured: microtubule binding, green; CENPE binding, blue; NudE binding, purple, leucine zipper region light yellow; Nup133 binding, light orange; Bub1 binding, maroon; nuclear localisation signal, red. **B** Clustal alignment of CENPF from orthologous species shows that residues Phe39 and Arg2147 are invariant across a broad evolutionary representation of organisms, whereas the other residues impacted by a missense variant are less conserved.

Twenty truncating variants have been reported in the literature plus 39 in ClinVar as pathogenic or likely pathogenic. Of these, 31 are nonsense variants and 28 are frameshifts. Four variants lie in essential splice site regions, and five variants are missense (Fig. 2A).

The majority of identified variants are truncating, with the position of all (except one) likely to trigger nonsense-mediated decay (NMD), and therefore be null alleles (Fig. 2A). Only a few variants have been seen in more than one family, with most being new variants identified. This observation could reflect sequence contexts where frameshifts might more easily occur due to polymerase slippage, or this possible pattern could just reflect the small number of cases reported thus far. The c.9280C>T, p.Arg3094* (NM_016343.4) variant lies in the final exon [20] and so is not expected to cause the *CENPF* transcript to undergo NMD, although RNA stability assays would be required to confirm this. The nonsense variant truncates the protein to remove the last 20 amino acids. Based on construct testing in vitro, the C-terminal 187 amino acids of CENPF are required for microtubule binding [27]. There are both conserved and less conserved patches across this 187 amino acid region, with the final six amino acids of CENPF strongly conserved. The effect of this terminal truncation on microtubule binding is thus not clear. Two cases in one family have been reported with this variant (in trans with an earlier nonsense variant, p.Glu582*); the first case died shortly after birth from multiple organ failure and the second case was terminated in utero with similar features [9].

Five pathogenic splice variants have been reported in *CENPF* (four in the literature, one in ClinVar) [10, 16, 19, 22]. One of the patients was homozygous for a variant predicted to disrupt the splice donor site in intron 7, c.1068+1G>A (NM_016343.4). RT-PCR analysis of parental leukocyte RNA showed that this variant caused skipping of exon 7, resulting in a frameshift (p.Glu289Valfs*33). While no splicing analysis has been undertaken to confirm effects of the remaining variants, all variants affect essential splice site positions, with very strong spliceAI scores that the canonical splice site is disrupted (lowest donor or acceptor loss score, 0.95).

Missense variants are much more rarely reported in *CENPF*, and this may be because of the recognised difficulty in assigning pathogenicity for many missense variants. In ClinVar, there are more than 450 missense variants classified as variants of uncertain significance. Of the five reported in the literature, all are reported in a homozygous state, but not all are strongly conserved or have high prediction scores (Fig. 2B). Functional evidence is available to support the c.117T>G, p.Phe39Leu variant [21]. In an experiment performed by the authors patient-derived fibroblasts were serum starved for 48 h and then studied for cilia characteristics. Compared to control cells, both the percentage of cells with cilia and the cilia length were both reduced in the patient-derived cells, supporting pathogenicity for the p.Phe39Leu substitution [21]. While in silico tools are generally supportive, no functional evidence is available to further support the pathogenicity of p.Thr729Ile, p.Leu849Val, p.Arg2147Trp, or p.Val1816Ala (this last variant was classified as likely pathogenic) [15, 19, 21].

### Clinical comparisons

**Fig. 3:**
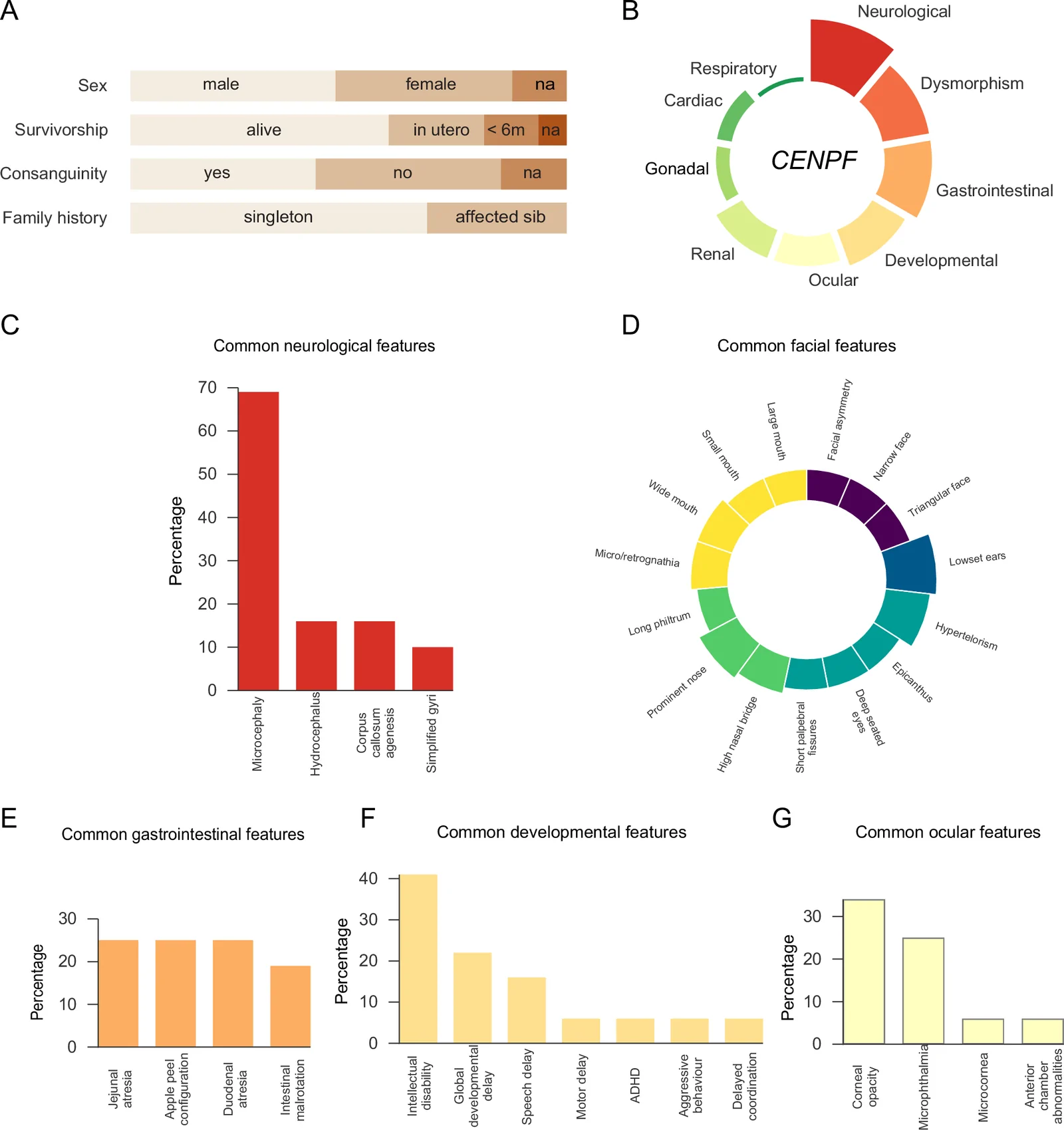
Clinical features associated with variants in *CENPF.* **A** Cohort proportions of sex, survivorship, consanguinity and family history. **B** Relative magnitudes of different affected systems. Most common features for different systems displayed as percentage of total cohort (*n* = 32): neurological (**C**), facial dysmorphism (**D**), gastrointestinal features (**E**), developmental (**F**), and ocular (**G**).

Thirty-one patients across 24 papers were identified, plus the individual reported here, with only those with a confirmed genetic diagnosis of Strømme syndrome (*CENPF* variants) included. Of the 32 patients, 15 patients were male, 13 were female and 4 didn’t report sex (Fig. 3A). Consanguinity was reported in 41% of patient’s families, 44% had no consanguineous family history and in 16% of cases it was not mentioned. Of all patients, 71% were reported as an individual and 29% were described alongside an affected sibling.

Gestational age at birth was reported for 20 patients, with 90% of these being born prematurely (*n* = 18). Nineteen of the 32 patients were alive at last follow-up, 7 were terminated during pregnancy or gestationally lethal and 4 died within 6 months of birth. Over 150 different clinical features were reported across the 32 patients, which we have categorised into neurological, dysmorphism, gastrointestinal, developmental, ocular, renal, gonadal, cardiac, respiratory and other (Fig. 3B). No single feature was present in every patient.

### Neurological phenotypes

Neurological features were the most prevalent, where 91% individuals had brain abnormalities (Fig. 3C). Of the 28 who reported brain abnormalities, the most common was microcephaly, reported by 75% (*n* = 21), followed by 18% with hydrocephalus (*n* = 5), 18% with corpus callosum agenesis/hypoplasia (*n* = 5) and 9% with simplified gyri (*n* = 3).

### Dysmorphic features

Fifty-four different dysmorphic features were reported in 21 individuals (66% of all patients), affecting all parts of the face (Fig. 3D). Of the individuals with dysmorphic features, 31% reported one of five different dysmorphisms of the eyes or eyebrows (*n* = 10), with the most common being hypertelorism (*n* = 4), deep seated eyes (*n* = 2), epicanthus (*n* = 2), and three cases with either short or downslanting palpebral fissures. A total of 31% of patients with dysmorphic features also reported dysmorphisms of the nose (*n* = 10), the most common being prominent or high nasal bridge (*n* = 3), prominent nose (*n* = 3), broad nasal root (*n* = 2), a broad, short nose (*n* = 2) or a long philtrum (*n* = 2). Ten patients were reported to have general face or head dysmorphic features (31%), including narrow face (*n* = 3), facial symmetry/microsomia (*n* = 2), pointed chin (*n* = 2), or triangular face (*n* = 2). Two cases had cleft palate.

Forty three percent of individuals reporting dysmorphic features had mouth or lip differences (*n* = 9), the most common being wide mouth (*n* = 3) and micro or retrognathia (*n* = 3). Eight reported ear dysmorphism (30%), of which 75% were low-set ears (*n* = 6).

### Gastrointestinal features

Of the 32 patients, 22 were reported to have a gastrointestinal issue (69%), 19 of which had some type of intestinal atresia (59% of all patients) (Fig. 3E). Jejunal atresia, duodenal atresia and apple-peel atresia were equally common (*n* = 8 each). Six individuals had multiple atresia types (19%), most commonly either jejunal or duodenal atresia in combination with apple-peel atresia. Seven patients were reported to have malrotation (22% of all patients), affecting the intestines (*n* = 6) or colon (*n* = 1). Various other gastrointestinal symptoms were identified in one patient each, including hyperechogenic bowel or partial bowel obstruction.

### Developmental disorders

A total of 17 patients were reported to have some type of developmental or behavioural disorder. However, 12 of the 32 cases would not have been evaluated for developmental delays, as cases were either gestationally lethal, had terminations of pregnancy or had died by 6 months post birth. If we exclude these cases from this category, 85% of patients had some form of developmental or behavioural disorder. The most common types were intellectual disability affecting 65% (*n* = 13), global developmental delay affecting 35% (*n* = 7), 25% with reported speech delay (*n* = 5), and 15% with motor delay (*n* = 3) (Fig. 3F). ADHD, aggressive behaviour, and coordination and problem-solving problems were each reported in two patients (10%).

### Ocular features

Fourteen of the 32 patients were reported to have an ocular impact (44%). The most common was any type of corneal clouding affecting 79% of patients with ocular involvement, including Peters anomaly and sclerocornea (*n* = 11) (Fig. 3G). Microphthalmia was commonly seen, present in 57% of those with ocular abnormalities (*n* = 8). Abnormal cornea size (*n* = 2) and anterior chamber abnormalities (*n* = 2) were each reported in 14% of cases. Six different ocular disturbances were reported in one patient each, these were blue sclera, irregular pupils, buphthalmia, iris atrophy, small optic disk, esotropia and iris stromal hypoplasia.

### Renal abnormalities

A total of 14 patients, or 44% were noted to have renal abnormalities. Of these patients, 57% had bilateral renal hypoplasia (*n* = 8) but eight other renal abnormalities were reported across the 14 patients including absent kidney (*n* = 2), hydronephrosis (*n* = 1) and horseshoe kidney (*n* = 1).

### Genital, cardiac and respiratory phenotypes

Six individuals had retracted testes, making up 40% of all males affected. Seven individuals have cardiac defects (22%). Of these patients, 86% had atrial septal defect (*n* = 6), but six other types of defects were also noted, including 29% with bicuspid aortic valve (*n* = 2). Two patients were reported to have respiratory issues (6%). One patient had bronchiolitis and a second had alveolocapillary lung dysplasia [9, 21].

### Other features

Variable other features were reported, spanning a wide range of systems affected and generally only identified in one person. Two cases had accessory spleens (*n* = 2) or hypotonia (*n* = 2), and two cases have preaxial polydactyly. One case had platyspondyly, and one individual had with upper thoracic kyphoscoliosis.

## Discussion

For rare developmental disorders, the standard approach for identifying the genetic variants has quickly become high throughput sequencing, through whole exome or genome sequencing (WES/WGS). Clinical features play an integral role in both considering candidate variants/genes, and in the formal classification of pathogenicity for variants. However, the hypothesis-free approach of WES/WGS has clearly led to the expansion of clinical features associated with variants in specific genes, as well as highlighting the inter-individual phenotypic variability that can be present. Following the initial report of a new gene-clinical association, and subsequent confirmatory case reports, it remains important to collate and summarise the clinical and molecular information to help guide efficient interpretation of newly identified variants for affected individuals.

Strømme syndrome, caused by biallelic variants in *CENPF*, is associated with intestinal atresia, neurological abnormalities and ocular abnormalities. Our review of the published case reports finds that these features are common, but variable, with no consistent feature present in any of the genetically diagnosed cases reported. Microcephaly was present in 66% cases, atresia in 59%, and ocular abnormalities in 44% of cases. Facial dysmorphism is just as common as the gastrointestinal or neurological issues, but features are very variable, with no clear facial gestalt to aid in diagnosis. In considering the triad of neurological, gastrointestinal and ocular abnormalities, the cohort is equally divided as to whether individuals are reported to have one, two, or three of these systems. The one case with none of these systems affected was a termination of pregnancy at 29 weeks, so a full assessment may not have been possible [19]. This case was homozygous for a missense variant (p.Leu849Val), which remains as a VUS. Some cases were identified from large cohort screening (for example, diverse congenital malformations or families with a history of consanguinity), where clinical information presented is more limited than individual case series, which could limit the information presented here. There does not appear to be any genotype-phenotype correlation, such as splicing or missense variants causing a less severe phenotype, or fewer systems involved.

Strømme syndrome is commonly characterized as a ciliopathy, based on functional information in the original disease gene identification [10]. In human cells, CENPF was detected in the basal body, with reduced cilia presence and length in patient-derived fibroblasts [10, 21, 28]. A zebrafish model also causes ciliopathy phenotypes of hydrocephalus, axis curvature defects and pronephric cysts [10]. Murine embryonic fibroblasts derived from a genetic knockout also observed consequences to cilia, though only in number of cells positive for cilia staining, and not cilia length. Broader defects also impact focal adhesion dynamics, cell migration, indicating severe effects on microtubule dynamics [29]. However, as more cases harbouring *CENPF* variants have been reported, the phenotype doesn’t strongly align with ciliopathies as first indicated. While renal cysts are a cardinal feature in ciliopathies [30], this was only observed in one case with *CENPF* variants [17], while renal hypoplasia, not typical in ciliopathies, was a more common feature, in 9/40 patients (23%). No retinopathy has been reported, and only 2/32 cases have polydactyly [9, 20]. Given the number of roles ascribed to CENPF in the cell, it may be that disruptions to the other functions, beyond a ciliogenesis role, contribute to the broad phenotypes observed.

## Supplementary information

Supplemental Table 1
